# Reawakening the Developmental Origins of Cancer Through Transposable Elements

**DOI:** 10.3389/fonc.2020.00468

**Published:** 2020-05-05

**Authors:** Chiemi F. Lynch-Sutherland, Aniruddha Chatterjee, Peter A. Stockwell, Michael R. Eccles, Erin C. Macaulay

**Affiliations:** ^1^Department of Pathology, Dunedin School of Medicine, University of Otago, Dunedin, New Zealand; ^2^Maurice Wilkins Centre for Molecular Biodiscovery, Auckland, New Zealand

**Keywords:** transposable elements, development, cancer, epigenetics, onco-exaptation, de-differentiation

## Abstract

Transposable elements (TEs) have an established role as important regulators of early human development, functioning as tissue-specific genes and regulatory elements. Functional TEs are highly active during early development, and interact with important developmental genes, some of which also function as oncogenes. Dedifferentiation is a hallmark of cancer, and is characterized by genetic and epigenetic changes that enable proliferation, self-renewal and a metabolism reminiscent of embryonic stem cells. There is also compelling evidence suggesting that the path to dedifferentiation in cancer can contribute to invasion and metastasis. TEs are frequently expressed in cancer, and recent work has identified a newly proposed mechanism involving extensive recruitment of TE-derived promoters to drive expression of oncogenes and subsequently promote oncogenesis—a process termed onco-exaptation. However, the mechanism by which this phenomenon occurs, and the extent to which it contributes to oncogenesis remains unknown. Initial hypotheses have proposed that onco-exaptation events are cancer-specific and arise randomly due to the dysregulated and hypomethylated state of cancer cells and abundance of TEs across the genome. However, we suspect that exaptation-like events may not just arise due to chance activation of novel regulatory relationships as proposed previously, but as a result of the reestablishment of early developmental regulatory relationships. Dedifferentiation in cancer is well-documented, along with expression of TEs. The known interactions between TEs and pluripotency factors such as NANOG and OCTt4 during early development, along with the expression of some placental-specific TE-derived transcripts in cancer support a possible link between TEs and dedifferentiation of tumor cells. Thus, we hypothesize that onco-exaptation events can be associated with the epigenetic reawakening of early developmental TEs to regulate expression of oncogenes and promote oncogenesis. We also suspect that activation of these early developmental regulatory TEs may promote dedifferentiation, although at this stage it is hard to predict whether TE activation is one of the initial drivers of dedifferentiation. We expect that developmental TE activation occurs as a result of the establishment of an epigenetic landscape in cancer that resembles that of early development and that developmental TE activation may also enable cancers to exploit early developmental pathways, repurposing them to promote malignancy.

## Introduction

Epigenetic modifications drive the transition from a single totipotent cell to an entire organism made up of a multitude of cell types. The three dimensional (3D) genome dictates cell fate through regulating gene expression. The transcriptional hierarchy consists of topologically associated domains (TADs), which demarcate higher order chromatin domains and establish and maintain regions of interaction and inactivity (1). Histone modifications regulate chromatin structure on a more local level and have a direct effect on transcription through the establishment of regions of “open” euchromatin and “closed” heterochromatin (2). DNA methylation is one of the most widely studied epigenetic mechanisms and is most well-known for its role in the silencing of transcription, particularly in the establishment of stable long-term repression. However, we are becoming increasingly aware of the interplay between DNA methylation and histone modifications, and the critical role that they play in both development and disease (3). Therefore, epigenetics not only plays a key role in development through determining cell fate, but also underlies many pathologies.

Epigenetic profiling of tumors has uncovered a complex epigenetic landscape of cancer cells and identified epigenetic alterations which are drivers of malignancy (4). However, despite recent progress in the field of cancer epigenetics, cancer remains one of the most elusive and devastating diseases. There are extensive epigenetic and functional similarities between early developmental stages and cancer. The placenta provides a unique window into early embryonic stages and shows further striking similarities to tumors in terms of its ability to invade and immunosuppress. It is known that epigenetic reprogramming of the extra-embryonic lineage mirrors the somatic transition to cancer (5). Dedifferentiation is recognized as a hallmark feature of cancer cells (6). This enables a resulting phenotype of proliferation, self-renewal and a metabolism reminiscent of embryonic stem cells (ESCs). There is also compelling evidence to support the idea that dedifferentiation of tumours can contribute to invasion and metastasis (7).

TEs have for many years been implicated in tumorigenesis and are increasingly being recognized as critical developmental regulators. TEs have facilitated vast diversification of vertebrates through donating novel species- and tissue-specific regulatory elements (8, 9). Enhancer and non-coding RNA (ncRNA) elements are considerably enriched for TE sequences, specifically those that have tissue-specific roles in ESCs and the placenta. To this end, it is now known that a large proportion of *OCT4, NANOG*, and *CTCF* binding sites in ESCs exist within TE sequences (10). Moreover, transcriptionally active TEs have also been implicated in distinguishing TAD boundaries in human pluripotent stem cells (11). Genes derived from TEs also exist. Many of these genes are primarily expressed in the placenta and function in pathways that liken the placenta to cancer such as proliferation, invasion, apoptosis and immunosuppression (12). These placental-specific, transposon-derived genes are expressed in some cancers (13). A role for TEs in cancer is well-established. Initial work focused on insertional mutagenesis and how this influences cancer progression, however more recently another important TE cancer interaction has been identified. Termed “onco-exaptation,” this process refers to the use of TE-derived promoters to drive expression of oncogenes and subsequently promote oncogenesis (14). Several mechanisms have been proposed to account for the rise of onco-exaptation.

**Figure 1:**
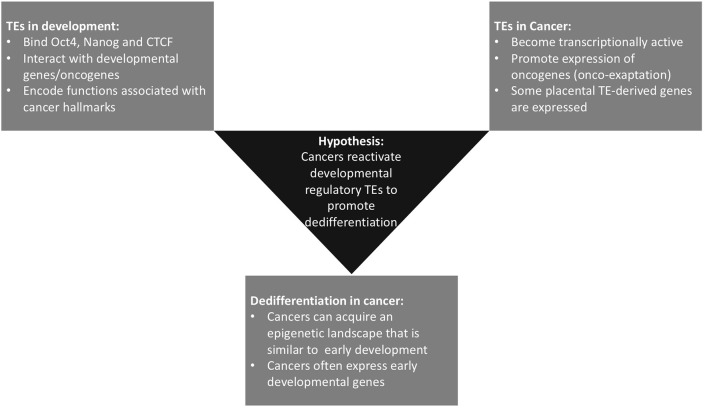
Summary of the three currently distinct fields within the literature that underpin our hypothesis. Some onco-exaptation events are associated with the epigenetic reawakening of early developmental TEs to regulate expression of oncogenes and promote oncogenesis. These TE-oncogene interactions either drive dedifferentiation or become reactivated as a consequence of dedifferentiation-associated epigenetic changes.

This hypothesis and theory article focuses on exaptation of TEs, providing examples of the critical roles of TEs in regulating development of the embryo and placenta and their function in processes that are also hallmarks of cancer in this context. We also discuss literature on TE expression in cancer, in the form of both onco-exaptation and expression of retrotransposon-derived genes in cancer. The evidence for dedifferentiation as a hallmark of cancer is also explored from the perspective that dedifferentiation-associated epigenetic changes that occur in cancer may facilitate activation of early developmental TEs and promote further dedifferentiation. Finally, based on the evidence discussed, we propose the novel hypothesis that onco-exaptation events can arise as a consequence of dedifferentiation-associated epigenetic changes, resulting in reactivation of these early developmental regulatory TEs. We hypothesize that these TE-gene regulatory relationships enable cancers to exploit developmental pathways, which are critical during early development, but when the developmental pathways are reactivated in cancer this reawakens regulatory elements and networks that have been established in early embryonic and extra-embryonic lineages, thus aberrantly facilitating cancer cell growth and survival (Figure 1). We provide insights into the potential impact of this on the diagnosis and treatment of cancer.

## TEs Contribute to Species-Specific and Tissue-Specific Regulatory Elements

### TEs as Drivers of Evolution

An intriguing phenomenon, which has been observed in the placenta, ESCs and cancer, is the loss of methylation at some TEs (15–17). These repetitive DNA sequences constitute 50% of the mammalian genome (18). Transposon activity has not only increased the size of eukaryote genomes throughout evolution, but has also contributed to the development of new gene networks and regulatory elements (19, 20). Faulkner et al. reported that up to 30% of all cap-selected human transcriptional start sites are found within TE sequences. Moreover, they identified considerable enrichment of tissue-specific transcripts within TE initiated sequences, suggesting that TEs are tightly spatially and temporally regulated (21). The origin of TEs as parasitic DNA elements has resulted in species developing mechanisms to silence TE transcription and minimizing the potentially deleterious impacts of these elements through transposition. DNA methylation and repressive chromatin marks play a key role in host-defense mechanisms against excessive TE activity. Alterations to such epigenetic regulators have been shown to directly impact transcription of TEs. Previous work has documented TEs as being almost always completely silenced by DNA methylation in healthy somatic tissues (22, 23). However, many evolutionarily older TEs in the genome have lost their ability to transpose or retro-transpose due to accumulated mutations in their replicative regions. As a result, in most cases it is only evolutionarily young elements that are actively transposing (24). Moreover, it is now known that TE transcription does not always result in transposition. Previously, most work involving TEs grouped them into classes and investigated genome-wide patterning rather than TE regulation at the individual element level. This likely contributed to the misconception that TEs are always silenced in healthy tissue. Indeed, TEs are highly abundant within mammalian genomes, and it is likely that the majority are methylated. However, there is now irrefutable evidence that some TEs are expressed and function as important genes and regulatory elements, particularly during early human development (8).

After fertilization, the early embryo undergoes epigenetic reprogramming, characterized by extensive demethylation (25). During this reprogramming, some TEs maintain a methylated state, similar to the maintenance of methylation at imprinted loci during global demethylation. Other TEs lose methylation initially but become remethylated through recruitment of the *de novo* methylation machinery (26). However, a number of TEs remain unmethylated and become transcriptionally active at this early stage. Many of these transcriptionally active TEs have been shown to regulate key pluripotency or totipotency factors (9). Recent work by Jonsson et al. showed that deletion of DNMT1 in human neural progenitor cells resulted in global loss of DNA CpG methylation. Amongst this they observed transcriptional activation and chromatin remodeling of evolutionarily younger (younger than 12.5 million years/hominoid specific) LINE-1 elements, whilst older LINE-1 elements remained silenced. Moreover, they found that the active LINE-1s functioned as alternate promoters for many protein coding genes with neuronal specific functions (27). These authors are not the first to implicate LINE-1s in development and disease (28, 29). Altogether this evidence supports the role of methylation in regulating TE expression and supports that some TEs escape methylation in a tissue-specific manner and have been co-opted to perform important functional roles.

The repetitive nature of TEs means they are notoriously hard to quantify, likely contributing to difficulty in the identification of functionally important elements. TE expression was previously considered to be a result of transposition, and detrimental to the host. Many studies and TE analysis tools grouped TEs into subfamilies when quantifying expression in order to minimize the issues with short RNA-sequencing reads mapping to multiple locations in the genome with high sequence similarity (30–32). However, this approach has resulted in a limited understanding of the locus level expression and regulation of TEs. Recent work addressing the issue of multimapping reads when analyzing highly repetitive TE sequences with short sequencing reads has revealed that many TEs are expressed in a locus-specific manner (33). As such, reads from TEs are often derived from a low number of highly expressed genomic loci, rather than from many loci that are lowly expressed. This supports the notion that some TEs are regulated in a similar manner to other genes and regulatory elements, challenging previous notions that they are parasitic DNA elements, transcribed purely for the purpose of transposition and propagation throughout the genome.

**Figure 2:**
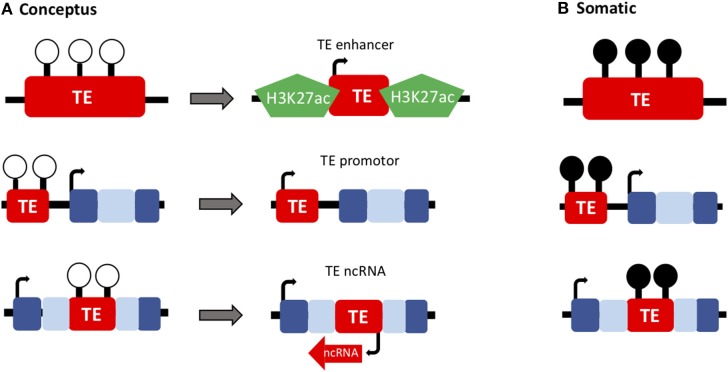
TEs have important developmental roles in the conceptus. **(A)** The epigenetic reprogramming that occurs during early embryonic development (loss of DNA methylation; white lollipops) has enabled the recruitment of TEs to function as genes and regulatory elements throughout evolution (such as TE-derived enhancers, promoters and ncRNAs) in the extra-embryonic (placenta) and embryonic lineages. **(B)** These developmentally important TEs are often methylated in healthy somatic tissues (black lollipops).

The idea that genes are less tolerant to change, and therefore it is often changes to regulatory networks that drive evolution, is becoming well-supported (34). This has been described in tissues such as the brain, which has diverged rapidly throughout mammalian evolution, particularly in primates (35). Moreover, there is evidence to support this model in placental evolution (36). There are huge amounts of structural and functional diversity of the placenta across different placental mammals. In the literature, there is widespread support that recruitment of TEs has helped to facilitate placental evolution, through enabling the establishment of novel, tissue-specific gene regulatory networks (37–41). This evidence now goes beyond the placenta, with significant evidence to support that TEs function as tissue-specific regulatory elements in a number of different tissues, particularly embryonic stem cells (29, 42–44). The role of TEs as critical mediators of pluripotency in the early embryo is now well-documented both through directly regulating transcription and through altering 3D chromatin structure (Figure 2). There are currently significant discrepancies within the literature regarding nomenclature for TEs and distinguishing between surreptitious TE expression resulting in transposition, and TE expression that is vital for normal cellular function.

### Methylation of TEs in the Placenta

The placenta is known for harboring unmethylated transposons (45), though it remains unknown how some of these sequences evolved to escape epigenetic silencing in a placental-specific manner. It is thought that the globally hypomethylated state of the placenta may have allowed for transposon sequences to become active and that this may have facilitated co-option of these elements (46). The transient nature of the placenta as an organ is likely to have minimized the potentially deleterious effects of transposon activity, as the placenta only exists for a period of 9 months in humans and does not persist into adulthood (47). Previously hypomethylation of retrotransposon sequences in the placenta was considered to occur non-specifically as a result of the hypomethylated state of the placenta (48). However, Chatterjee et al. carried out a genome-wide methylation comparison between placenta and neutrophils and discovered that loss of placental methylation is in fact more pronounced at non-retroelement containing sequences (49). This work suggests that loss of methylation at retrotransposon sequences in the placenta occurs with some sequence specificity and may correspond with sequences that have acquired functional roles in placental development.

### Functional TEs in the Placenta

TEs have significantly contributed to rapidly evolving gene regulatory networks during mammalian evolution both in *cis* and in *trans*. This is likely due to the existence of regulatory motifs, which were very similar to transcription factor binding sites within TEs, which facilitated the co-option of these regions into host gene regulatory networks. Recruitment of TE sequences to function as *bona fide* genes and regulatory elements has been termed exaptation. These genes and regulatory elements which contain transposon sequences can also be referred to as transposon-derived and transposon-regulated genes. Some of the first examples of exaptation events were identified in the placenta. These events were likely enabled by the hypomethylated state of the placenta. Chuong et al. investigated placental-specific enhancers in rat and mouse and found that these elements were highly enriched for endogenous retroviral (ERV) sequences, and that retroviral recruitment was enriched in tissue types with lower levels of DNA methylation (36). This suggests that lower levels of DNA methylation facilitated the recruitment of normally silenced TEs, which enabled the evolution and diversification of new regulatory networks. A number of placental-specific promoter elements in the human genome are derived from ERV sequences (50). In some cases, these are alternate promoters to the somatic promoter, giving rise to placental-specific transcripts (51). *KCHN5* is a voltage-gated potassium channel, which has a diverse range of functions. It is expressed in a number of different somatic tissues; however, it is the placental-specific isoform that is promoted by a TE (12). *PTN*, another placental TE-derived gene has a critical role in initiating angiogenesis and also functions in cell differentiation in the placenta (52). The human placenta is known for expressing more transposon-derived promoters than any other tissue (45, 53). These promoters have been shown to regulate expression of functionally important placental genes, and are thought to have made a significant contribution to the rapid diversification in form and function seen across placental mammals (54). The function of these genes in the placenta in processes which are also observed in cancers supports that this function may be conserved in cancer. And as such these genes may be recruited by cancer cells in order to facilitate tumor progression (55–57).

### TEs as Developmental Regulators

Further work has demonstrated that TEs provide widespread contributions to tissue-specific and species-specific gene regulatory networks, not just in the placenta (58). Regulatory regions of the genome are characterized by specific chromatin configurations, including DNA methylation and histone modifications, which act to either facilitate or block interactions with transcription factors and subsequently control gene expression (59). Long terminal repeat (LTR) elements are strongly associated with enhancer histone marks (H3K27ac, H3K4me1) and frequently contain functional transcription factor binding sites. Sixty-six percent of predicted LTR enhancers acquire active regulatory histone marks in a cell- and species-specific manner (44). Moreover, they appear to be linked with genes that function in regulatory pathways that correspond with specific cell types. Non-coding RNAs are increasingly being recognized as regulatory elements. However, it remains unclear how abundant these elements are and their specific function (60). Some have been shown to target mRNAs resulting in their degradation, while others are involved in directly regulating transcription (61). Again, these ncRNAs frequently contain TE sequences and present another example of regulatory elements which have evolved from TE elements (42). Categorization of long-intergenic non-coding (linc) RNAs has revealed that 83% contain a TE, and TE sequences make up 42% of lincRNA sequence. Some TE-derived lincRNAs show stem cell-specific expression, which is consistent with the idea that these elements have high tissue specificity and are biased toward early developmental stages (62).

**Figure 3:**
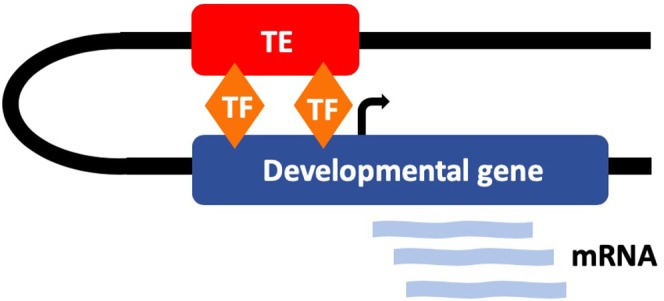
TEs have evolved to function as tissue-specific regulatory elements that regulate early development. Many TEs contain transcription factor (TF) binding sites for key developmental regulators (such as *OCT4* and *NANOG*) and interact with developmental genes.

The occurrence of functional TEs driving early development is highlighted by the key transcriptional regulators, *NANOG, OCT4*, and *CTCF* in human and mouse ESCs (10). *OCT4* and *NANOG* are two of the four Yamanaka factors that are required for the reprogramming of fully differentiated cells to pluripotency and play an essential role in the maintenance of pluripotency in stem cells (63, 64). CTCF is a critical transcription factor which has been shown to both activate and repress expression and recruit other factors to maintain chromatin boundaries (65). Interestingly all three of these transcription factors have been shown to be dysregulated in various cancers (66–68). Kunarso et al. found that the protein binding sites of OCT4 and NANOG are highly divergent between mouse and human with <5% being homologous. Moreover, their work uncovered that 25% of the binding sites in both species were donated by TEs (10). This provides notable evidence in favor of the role of TEs in rewiring the transcriptional network of ESCs (Figure 3). Furthermore, it highlights the high level of diversity in the regulation of highly evolutionary conserved developmental regulators. It is likely that the tissue and species-specific nature of these regulatory networks, along with the misconception that TEs were purely parasitic “junk DNA,” contributed to their being previously overlooked as regulators. However, there is now substantial evidence to dismiss this idea. Due to the abundance of TEs in the genome, the majority are very likely non-functional, but it is becoming apparent that a significant proportion are, in fact, critical regulators of genome function, particularly during early development (69). It is plausible that the unmethylated state of some TEs during early development has facilitated their recruitment to function as regulators during this early stage of life.

Some TEs have been implicated in the establishment of topologically associated domains (TADs) in human pluripotent stem cells (hPSCs). TADs are large regions of chromatin that are separated by boundaries. 3D interactions occur at a much higher frequency within TADs than between them (70). Consequently, TADs are known to be critical in mediating 3D interactions between promoters and enhancers and thus can be important in cell type specificity (71). Whilst some TAD boundaries are often conserved between different species and cell types of the same species, some are cell type- or species- specific. Some TADs are reconfigured during differentiation owing to the different regulatory networks required in differentiated vs. pluripotent cells (72). Moreover, TADs have been shown to be disrupted in various malignancies (73). Zhang et al. recently interrogated the remodeling of the 3D genome during cardiomyocyte differentiation in humans and discovered a novel role for the endogenous retrovirus subfamily HERV-H in the establishment of TADs in hPSCs. They demonstrated that deletion of the HERV element at a TAD boundary eliminated the boundary and reduced the transcription of upstream genes. They also showed that insertion of HERV-H could introduce a new TAD boundary (11). Based on the known role of TAD boundaries in insulating interactions between TADs, it is plausible that HERV-H elements either have insulatory capacity, or are able to recruit factors that regulate TAD boundary formation, such as CTCF. Taken together, this provides support for the role of TEs as regulators in *cis* and in *trans* during early human development.

## The Roles of TEs in Cancer

Expression of transposons has long been implicated in cancer development (74). The documented silencing of these elements in healthy somatic tissues led to the assumption that transcription of TEs was deleterious. Indeed, transposon activity can interrupt gene expression and function by inserting into the promoter or coding sequence. Additionally, the repetitive nature of these sequences can result in incorrect recombination events and lead to translocations, deletions and insertions (16, 75). The predominant mode of retrotransposon-driven oncogenesis was considered to be by these mechanisms, resulting in genomic instability (76, 77). Sporadic evidence exists for *de novo* insertions contributing to oncogenesis however this was not extensive enough to account for the entirety of this phenomenon. An upregulation of TEs on a genome-wide scale as a result of loss of DNA methylation has also been documented. This has been linked to changes in response to immune therapies as a result of activation of the innate immune response (78).

### Placental TE-Derived Genes in Cancer

Some known placental genes and regulatory elements derived from TEs have been documented to lack methylation and be expressed in cancer. Members of our group previously identified six placental-specific transcripts of genes that are hypomethylated in melanoma in comparison to their methylated state in somatic tissues (12). The methylation changes were correlated with expression of these transcripts in melanoma cells (13). A number of these genes have also been identified as candidate oncogenes due to their expression in various other cancer tissues. The functions of these TE-derived genes in the placenta are also key hallmarks of cancer cells (i.e., invasion, immune modulation, growth/proliferation, etc.), suggesting that these genes may retain this function when reactivated in cancer. As such, these genes may be hijacked by cancer cells in order to facilitate tumor progression (55–57). This work demonstrates that regulatory TEs that have key placental functions can become reactivated in cancer. Some examples of these TE-promoted or TE-derived placental genes (*PEG10* and *Syncytin*) have been identified independently to function as oncogenes in human cancers (79, 80).

### Onco-Exaptation: TE-Derived Promoters Driving Oncogene Expression in Cancer

**Figure 4:**
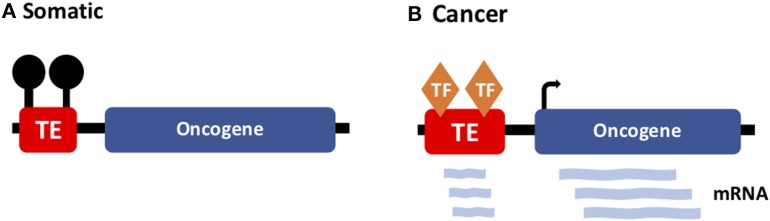
TE-driven oncogene expression in cancer. **(A)** In somatic tissues, TEs (with regulatory potential) are silenced by DNA methylation. **(B)** In cancer, onco-exaptation occurs when somatically-dormant TEs lose methylation and function to drive expression of oncogenes.

More recently, a new TE-cancer interaction has been identified—termed “onco-exaptation”—which involves the recruitment of regulatory motifs within TE sequences to drive oncogene expression (Figure 4). Initially, these events were identified in discrete cases specific to one particular subtype of cancer. In 2016, Babaian et al. published work characterizing an onco-exaptation event whereby an endogenous retroviral LTR element was driving expression of *IRF5* in Hodgkin's lymphoma. They demonstrated that the LTR-IRF5 chimeric transcript was specific to Hodgkin's lymphoma cell lines and was not present in any healthy B-cell controls (81). Further studies have identified examples of onco-exaptation in melanoma, colorectal cancer and lymphoma (14). More recently Jang et al. undertook a genome-wide search for onco-exaptation events across 15 different cancer types and demonstrated the breadth of these events in cancer. They also established that deletion of the TE was sufficient to silence the related oncogene. Finally, they altered methylation at the same TE using CRISPR-cas editing, which resulted in modulated promoter activity, suggesting a role for methylation in facilitating onco-exaptation. Their findings uncover the extent of onco-exaptation events across a range of different cancers and bring onco-exaptation into the spotlight as an important contributor to oncogenesis (82).

TE-derived lncRNAs have also been implicated in tumorigenesis, as reviewed by Babian and Mager (14). Strikingly, many lncRNAs that have been implicated in cancer thus far are suspected to interact with genes that are developmentally important and frequently oncogenic (83). To date, the literature on onco-exaptation has proposed that these regulatory relationships are cancer-specific due to methylation of the interacting TE in the corresponding somatic tissue. Two prominent models for onco-exaptation are the de-repression model and the epigenetic evolution model. The de-repression model predicts that molecular changes which occur during oncogenesis activate TEs and result in the establishment of TE-driven expression of oncogenes (84). There is support for this model from observations that specific factors must be present in order for a given TE-derived promoter to become active. However, it is yet to be determined what factors specifically activate TEs in cancer, and why a given locus becomes active, given that identical TEs occurring throughout the genome usually do not become active. In contrast to the de-repression model, the epigenetic evolution model proposes that there is a high level of epigenetic variability both between specific TE loci, and at the same locus within a population of cells. This variability can induce novel regulatory interactions, which are increased in cancer. For example, at any given time in a cell, some TE loci will be unmethylated and therefore have the potential to acquire enhancer/promotor activity. Unlike the de-repression model, the epigenetic evolution model proposes that a pathogenic state (such as cancer) is not required for the transcription of TEs, but instead, the epigenetic state is ultimately responsible for permitting TE transcription. When a TE-regulatory event arises that confers a selective advantage to that cell, its clone would increase in frequency within a tumor population, resulting in a population of cells expressing the given TE, and corresponding oncogene (14).

**Figure 5:**
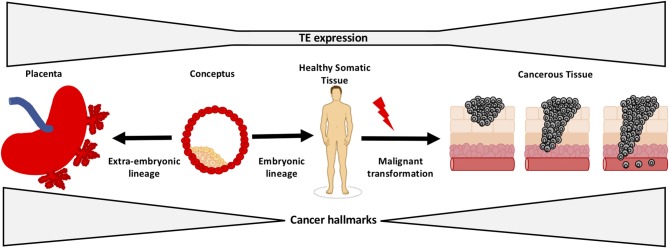
TEs regulate early development and tumorigenesis. Tissue-specific TEs function to regulate early development and are silenced by DNA methylation in healthy somatic tissues. It is known that some TEs become activated and drive expression of oncogenes in human cancer. The evidence suggests that cancers adopt early developmental TEs from the placenta/early embryo and utilize them to activate processes that are critical in the early conceptus, but drive pathological hallmarks of cancer.

Proposed models for the formation of onco-exaptation events are highly plausible, and previous work supports that both are likely to contribute to a degree. However, the suggestion that regulatory relationships between TEs and oncogenes in cancer is novel is often based on investigation of the corresponding somatic tissue, in which these relationships are not present. To the best of our knowledge, no one has yet profiled the onco-exaptation candidates in either hESCs or in the placenta. We expect that some TE–cancer regulatory relationships are not novel to cancer, but arise due to the re-establishment of an epigenetic state that resembles early development and likely promotes further dedifferentiation (Figure 5). This idea could align with both of the existing models for onco-exaptation (the de-repression model and the epigenetic evolution model).

It is now clear that there is an interplay between TE activation, dedifferentiation and tumor progression. However, the question remains as to what extent TE activation promotes dedifferentiation, or vice versa. Based on the current literature, it is hard to draw conclusions about the initial inducers of dedifferentiation in cancer. There is increasing evidence supporting epigenetic events as having a fundamental role in inducing oncogenesis, sometimes in the absence of driver mutations. Feinberg et al. propose a framework for cancer epigenetics that involves epigenetic mediators, which are genes that become disrupted in the early stages of malignancy and induce an altered differentiation state during tumor evolution (85). Epigenetic mediators contribute to phenotypic plasticity and tumor progression and frequently overlap with genes involved in reprogramming (such as *OCT4* and *NANOG*). Given the substantial interaction between these transcriptional regulators and TEs during early development, and the known role of these transcription factors in promoting malignancy, it is likely that TEs mediate the transcriptional network of pluripotency factors in cancer as they do during development. Moreover, the recent identification of primate specific TEs in delineating TAD boundaries in stem cells supports the hypothesis that TEs may play fundamental roles in generating cell type specificity and in the facilitation of regulatory networks. The fact that some TAD boundaries are known to change in cancer highlights the possibility that early in oncogenesis, TE activation may be involved in facilitating changes in chromatin structure and therefore activation of alternate regulatory networks.

## Dedifferentiation in Cancer: Support for Activation of Developmental TES

### Evidence for Dedifferentiation in Cancer

Studying dedifferentiation in the context of induced pluripotent stem cells (iPSC) has enabled insights into the epigenetic signatures of dedifferentiation, and highlighted the tendency of some cancers to dedifferentiate during tumor progression (6). Moreover, incomplete reprogramming of somatic cells to iPSCs has been shown to result in malignant transformation (86), supporting that epigenetic signatures of reprogramming can drive cancer, even in the absence of the mutation profile often characteristic of cancer genomes. Dedifferentiation is considered to be a hallmark of cancer, however the mechanisms which induce dedifferentiation in a cancer microenvironment remain elusive, as does the complete picture of the genetic and epigenetic signatures (87).

The selective advantage of obtaining stem cell-like characteristics is obvious through the acquisition of unparalleled self-renewal and proliferative capacities. The trend of tumors to lose differentiation markers and reacquire an epigenetic landscape reminiscent of early developmental stages is well-supported (87). Unsurprisingly, this epigenetic reprogramming is correlated with expression of early developmental genes. Many of the transcription factors which induce cellular reprogramming, or are fundamental to maintaining a pluripotent state, are also potent oncogenes. Additionally, the tumor suppressor p53 has been shown to inhibit reprograming, as it does for tumorigenesis (88). *TP53* is one of the most frequently mutated genes in human cancer. Loss of p53 function coincides with loss of senescence and apoptosis pathways in response to cellular stress (89). It is thought that p53 can inhibit nuclear programming by induction of cellular senescence through activation of p21 (90). This is considered to be a major roadblock in the path to pluripotency and, as such, inactivation of p53 increases reprogramming efficiency (91). Taken together this provides further support for the overlapping mechanisms both promoting and preventing reprogramming and tumorigenesis.

It is known that ESCs have distinct properties from fully differentiated cells. Replicative immortality, increased proliferative capacity and a distinct metabolism are fundamental stem cell traits that can also be observed in cancer. Embryonic stem cells are capable of both self-renewal (to maintain a population of stem cells) and differentiation (92). Somatic stem cells also exist. However, these are further differentiated, and therefore can only give rise to a subset of somatic cells. Increased telomerase activity is another feature which is critical to the replicative immortality of stem cells. Telomeres are repetitive regions of DNA that flank chromosomes and function to maintain stability during replication (93). As cells undergo multiple mitotic events, their telomeres become degraded. Telomerase is capable of maintaining telomeres, however it becomes repressed in somatic tissues allowing for cells to enter senescence after a certain number of divisions. In embryonic stem cells, telomerase is activated and thus maintains telomere length, enabling unlimited replication. Interestingly, adult stem cells show an intermediate level of telomerase activity, corresponding to their higher replicative potential in comparison to fully differentiated cells (94). Increased telomerase activity is also a hallmark of cancer cells. The acquisition of a metabolism reminiscent of ESCs has also been observed in cancer. Termed “metabostemness,” these metabolic alterations are thought to render a cell more receptive to certain epigenetic changes, which ultimately facilitate dedifferentiation (95).

### Functional Similarities Between the Placenta and Cancer

The placenta shares a number of additional features with cancer, stemming from its function to sustain fetal growth while evading the maternal immune response. Both placental and cancer cells have the ability to invade and demonstrate increased proliferative abilities (96). Reduced cell death has also been attributed to the rapid growth of the placenta and cancer, notably due to resistance of apoptosis through expression of proteins, such as Survivin (97). Additionally, both tissues have the ability to initiate angiogenesis to establish a blood supply and facilitate growth. Immune evasion also plays a key role in both tumorigenesis and placentation (98). A key function of the placenta is disguising the developing fetus from the maternal immune response. This occurs through the dampening of the maternal immune system, and also though immunological disguise of the fetus by the placenta (99). The placenta is known to lack expression of most MHC class 1 molecules, and thus is less recognizable to the maternal immune cells. Notably, the placenta and tumors also express *PD-L1* and *galectin-9*, both of which are known to be immune modulators (41, 100). *PD-L1* expression is associated with a poorer prognosis for some malignancies as it functions to dampen the immune response to the tumor through inactivation of cytotoxic T cells. A current theory in the literature is that epithelial cancers may be an undesirable consequence of evolution of the invasive eutherian placenta. This proposes that placental immune editing switches have evolved to enable immunological disguise of the fetus by the placenta and that these same mechanisms become exploited by cancer cells, contributing to the increased incidence and lethality of epithelial tumors in placental mammals (101). It seems plausible that cancer cells would be more likely to reactivate innate immune suppression pathways that evolve *de novo* mechanisms, however further work is needed to confirm this idea. Nonetheless, immune evasion is fundamental for both tumor and placental growth, so it is unsurprising that shared mechanisms exist between these two tissue types.

Utilization of the epithelial to mesenchymal transition (EMT) is also critical in both placental and tumor growth. This occurs when epithelial cells show a switch to a mesenchymal phenotype resulting in increased motility through loss of cell-to-cell contact inhibition and increased invasive capacity (102). It is critical in development, particularly in the placenta but can also contribute to pathologies such as fibrosis and cancer progression (103). Cytotrophoblast cells of the placenta undergo this transition when differentiating into extra villous trophoblasts, and disruption of this process has been linked to placental pathologies, including pre-eclampsia (104). EMT contributes to cancer progression, specifically in facilitating migration of cells of the primary tumor to form metastatic tumors (105).

### Environmental Conditions Shared by the Placenta and Cancer

During early placentation the invading trophoblastic cells are exposed to severe hypoxic conditions. Hypoxia is also well-documented in tumors and occurs to varying degrees both spatially and temporally during tumorigenesis. The implications of hypoxic conditions are not fully understood. However, it has been shown that exposure to hypoxia can increase the rate of cell division in order to seek out an oxygen supply (106). Hypoxia can also induce hypomethylation in cancer cells, however the extent to which this occurs is unknown. The hypoxia inducible factor (HIF) is known to be expressed in both placental and tumor cells in response to low oxygen levels. HIF expression has downstream effects on a number of different pathways including metabolism, angiogenesis and immune modulation. Notably, temporal expression of PDL-1 in invading trophoblast cells has been linked to oxygen availability (100). HIF knockout mice demonstrate embryonic lethality owing to abnormal placentation. HIF dysregulation has also been observed in a number of cancers. A recent review by Macklin et al. highlights the similarities between the placental and cancer microenvironments, and discusses hypoxia and subsequent HIF expression as a potential link between some of the shared functions carried out by both tissues (106).

### Implications of Dedifferentiation in Cancer

**Figure 6:**
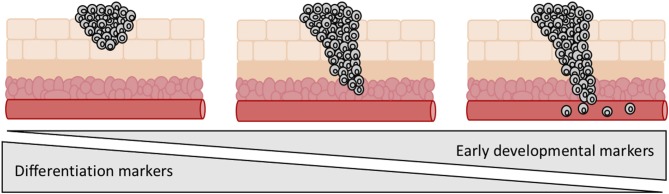
Expression of early developmental genes and loss of differentiation markers is linked to tumor progression. Aggressive metastatic tumors tend to be enriched for expression of early developmental genes and show a less differentiated morphology.

Studies have investigated the differentiation status of tumors in relation to invasion and metastasis with fascinating results. It is apparent that expression of differentiation markers declines as a cancer progresses and stem cell markers become more predominant (Figure 6). Tsoi et al. identified four distinct Melanoma subtypes that follow a differentiation trajectory ranging from a dedifferentiated group enriched for expression of invasive markers, to a differentiated melanocytic type (107). Melanoma demonstrates high levels of heterogeneity and plasticity and is known to dedifferentiate in response to cellular stress, often in the form of pro-inflammatory signaling (108). This is one mechanism that contributes to resistance to immunotherapies. Of further interest is that the dedifferentiated groups also demonstrate a more invasive phenotype. Murine models for melanoma have demonstrated that partial reprogramming results in a phenotypic switch to an invasive state. This switch was shown to be reversible, highlighting the ability of melanoma to adapt in response to external and internal stimuli (7). Tsoi et al. discovered that the dedifferentiated subtypes showed increased sensitivity to ferroptosis, highlighting this approach as a potential option to block dedifferentiation-facilitated resistance to immunotherapies (107). Similar trends have been uncovered in a number of other cancers. Aggressive metastasis-prone lung cancers show activation of early developmental genes as a result of malignant epigenetic reprogramming (109). Similarly in colorectal cancer, TGF-beta has been shown to contribute to dedifferentiation resulting in stem cell-like properties, which are linked to a poorer prognosis in patients due to recurrence and metastasis (110).

## Molecular Evidence for Dedifferentiation in Cancer

The functional similarities of ESCs, iPSCs, cells derived from the extra-embryonic lineage and cancer, combined with the intertwined relationship of epigenetics and cell fate, have prompted investigations into whether these cell types also share epigenetic features, particularly DNA methylation. Interestingly, large-scale methylation analysis in colon cancer (compared to normal colon) revealed that the majority of differentially methylated regions (DMRs) were not located within promoter-associated CpG islands—instead these DMRs frequently occurred up to 2 kb from the somatic promoter CpG island (111). These regions have been termed “CpG island shores.” Doi et al. found a similar trend when investigating DMRs in differentiated fibroblasts compared to reprogrammed iPSCs from fibroblasts. They localized methylation changes to CpG island shores that are now recognized as markers of development and dedifferentiation (112). Overall, this work demonstrated an overlap in differentially methylated CpG island shores that are important in pluripotency in both ESCs, iPSCs and in cancer cells. However, the specific function of these CpG island shores remains unknown. Interestingly, TE-derived promoter regions often exist upstream of the canonical promoter, and thus may overlap with these CpG island shores (and therefore fall within DMRs), however this idea needs further investigation.

### Shared Epigenetic Features Between the Placenta and Cancer

The majority of work comparing the methylation profile of early development with that of cancer has been done in the placenta. The placenta is globally hypomethylated in comparison to healthy somatic tissue. On average, it demonstrates a 22% reduction in DNA methylation compared to healthy somatic tissue, but is hypermethylated at known tumor suppressor genes (49). Notably, the placenta shows a DNA methylation landscape more similar to that of tumors than to other healthy somatic tissues. A recent paper by Smith et al. investigated global re-methylation of the early epiblast and extraembryonic lineage in mouse and showed two highly divergent methylation landscapes. Moreover, despite a global reduction in methylation, the extra-embryonic lineage acquires specific *de novo* methylation markings at a number of CpG island promoters which are associated with key developmental regulators. These same regions are recorded as being methylated in many human cancers. Based on this, the authors propose that during tumorigenesis, tumors may reacquire this developmentally encoded epigenetic landscape and subsequently a gene expression profile similar to that of the extraembryonic lineage (5).

Further work investigating the human placenta has shown that global hypomethylation along with hypermethylation at CpG islands characterizes the placental methylome. Nordor et al. provided the first evidence to suggest that the early placental methylome is more similar to that of tumors than the full term placental methylome. During the first trimester, the human placenta has additional hypomethylated blocks of a similar size and location to those of solid tumors. Moreover, these blocks are lost as the pregnancy progresses. Further analysis of these genomic regions has revealed that they encompass genes involved in pathways that are considered to be hallmarks of the placenta and cancer, such as EMT markers, immune modulators and inflammation (113). This result is intriguing because placental invasion reaches its peak at 12 weeks of gestation, suggesting that expression of key invasive genes would also be at a maximum during this stage. Taken together, these data support the idea that the shared functions of the placenta and cancer may be governed by similar epigenetic landscapes. Future work should investigate these common DMRs at a higher resolution and explore their functional significance.

## Discussion

In cancer, it is important to acknowledge the large degree of heterogeneity and the dysregulated nature of the cancer genome, which occurs as a result of both genetic and epigenetic aberrations. Nonetheless, malignant transformation is always accompanied by epigenetic changes, frequently involving methylation alterations. The patterns of methylation in cancer show striking similarities to both ESCs and the placenta. Of significant relevance, it is observed that common DMRs exist between ESCs, iPSCs, and cancer cells when compared to differentiated cells. Strikingly, these DMRs do not exist at the canonical promoter of these genes but are frequently located upstream in CpG island shores. DNA methylation is a key regulator of TE activity, and consequently, loss of methylation has been shown to be sufficient to activate TE expression. Furthermore, TEs driving onco-exaptation often exist upstream of the somatic promoter. TEs have made a substantial contribution to the evolution of early developmental species-specific regulatory networks and are known to regulate key developmental genes. The subsequent role of many of these genes as oncogenes provides further support for the idea that TEs promote tumorigenesis through reactivation of early developmental regulatory relationships.

**Figure 7:**
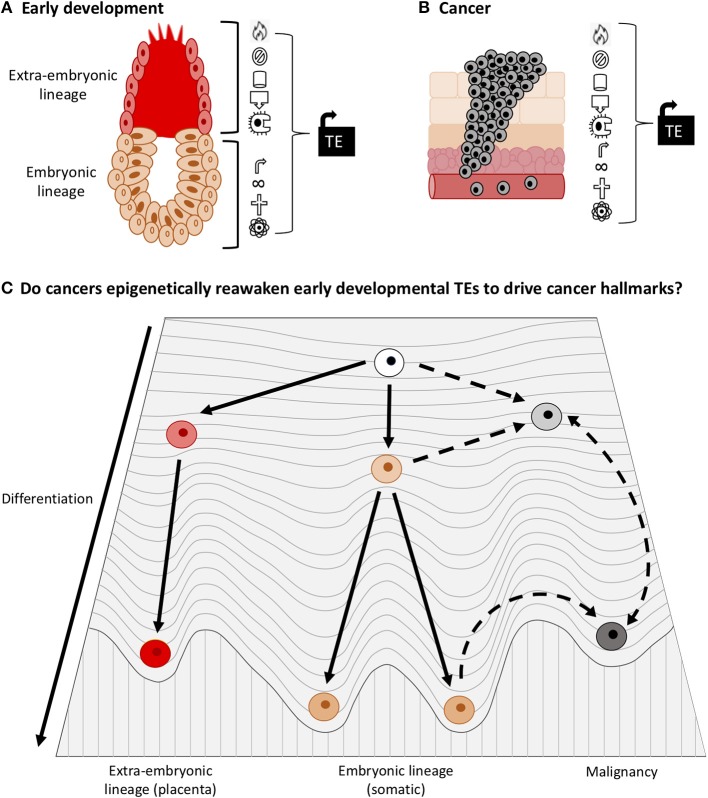
TEs play important roles in early development and cancer. **(A)** TEs drive processes in the early embryo and placenta that also occur in cancer (Cancer hallmarks such as proliferation, immune evasion, angiogenesis, and invasion). **(B)** TEs drive expression of oncogenes in cancer and thus promote cancer hallmarks. **(C)** Adaptation of Waddington's epigenetic landscape for development and cancer. Less differentiated cells have a higher entropy, more transcriptional “noise” and greater phenotypic plasticity than differentiated cells. Due to the documented roles of TEs in development and cancer, and the tendency of cancer cells to dedifferentiate, we surmise that some TE networks that drive early development become epigenetically reactivated in cancer. Accordingly, we raise the question: does TE activation enable cancer cells to dedifferentiate into a state of higher entropy (during which onco-exaptation can occur), or do dedifferentiation associated epigenetic changes facilitate activation of developmental TEs?

In cancer cells, the adoption of a dedifferentiated state is widespread, and many oncogenes have important developmental functions. It therefore appears that some onco-exaptation events are probably not novel alterations in cancer, but are in fact the re-awakening of early developmental regulatory networks through the establishment of an early developmental epigenetic landscape. To our knowledge, this concept has not been proposed explicitly. We believe that this hypothesis could align with both the de-repression and the epigenetic evolution models for onco-exaptation and further work is needed to establish the extent to which each drives onco-exaptation events. We propose that some TEs become activated as a result of molecular changes that occur during oncogenesis, which is in line with de-repression model. However, we also expect that, as a result of dedifferentiation in cancer, malignant cells have higher transcriptional “noise” and entropy, which is in line with Waddington's developmental epigenetic landscape model. This transcriptionally “noisy” state would create variability in the activity of TEs, which would enable the activation of TE-oncogene regulatory relationships, as proposed by the epigenetic evolution model (Figure 7). Currently there is no direct evidence to support our hypothesis that early developmental TEs become reactivated to contribute to dedifferentiation in cancer. However, due to the role of TEs in development and their interactions with pluripotency factors such as OCT4 and NANOG, along with the known activation of such factors in many cancers we believe this hypothesis warrants further investigation. Moreover, the well-documented epigenetic similarities between early development and cancer may create a state permissive for activation of early developmental TEs. These events involving TE-oncogene interactions may be fundamental drivers of malignancy and underpin oncogenic activation. Additionally, the origins of TE regulatory networks in early development and the absence of TE expression in healthy somatic tissues suggest that they could make appealing therapeutic targets.

In conclusion, the lack of a granular focus on TEs has biased investigations and delayed appreciation of TEs as key developmental regulators or as drivers of oncogenesis. Categorization of the abundance and function of TEs in normal development may help to provide information on their function in their native context (in the absence of the mutated and dysregulated background of cancer cells). A focus on locus-specific regulation of TEs, rather than group-wise expression and regulation at the subfamily level, would add clarity to these investigations. Investigating the role of TEs in early development and in cancer is likely to be of significant clinical relevance due to the known implications of TE expression and dedifferentiation in response to therapies. Henceforth, a shift in the way we view these two (currently separate) fields may provide novel insights into both fields, which would likely correspond to improved outcomes in the clinic.

## Author Contributions

CL-S, EM, and ME conceptualized the manuscript. CL-S wrote the first draft of the manuscript. AC and PS revised the manuscript and contributed ideas. ME and EM provided supervision, guidance, and manuscript editing and funding. All authors read and approved the final manuscript.

## Conflict of Interest

The authors declare that the research was conducted in the absence of any commercial or financial relationships that could be construed as a potential conflict of interest.

## Abbreviations

TEs: transposable elements
ESCs: embryonic stem cells
3D: Three-Dimensional
ncRNA: non-coding RNA
TAD: topologically associated domain
ERV: endogenous retrovirus
lincRNA: long-intergenic non-coding RNA
hPSCs: human pluripotent stem cells
iPSC: induced pluripotent stem cells
EMT: epithelial to mesenchymal transition
HIF: hypoxia inducible factor
DMRs: differentially methylated regions.
